# Influence of Test Specimen Geometry on Probability of Failure of Composites Based on Weibull Weakest Link Theory

**DOI:** 10.3390/ma15113911

**Published:** 2022-05-31

**Authors:** Rajnish Kumar, Bo Madsen, Hans Lilholt, Lars P. Mikkelsen

**Affiliations:** Division of Wind Energy Materials and Components, Department of Wind and Energy Systems, Technical University of Denmark, 4000 Roskilde, Denmark; boma@dtu.dk (B.M.); hali@dtu.dk (H.L.); lapm@dtu.dk (L.P.M.)

**Keywords:** Weibull distribution, strength, mechanical testing, unidirectional composites

## Abstract

This paper presents an analytical model that quantifies the stress ratio between two test specimens for the same probability of failure based on the Weibull weakest link theory. The model takes into account the test specimen geometry, i.e., its shape and volume, and the related non-constant stress state along the specimen. The proposed model is a valuable tool for quantifying the effect of a change of specimen geometry on the probability of failure. This is essential to distinguish size scaling from the actual improvement in measured strength when specimen geometry is optimized, aiming for failure in the gauge section. For unidirectional carbon fibre composites with Weibull modulus m in the range 10–40, it can be calculated by the model that strength measured with a straight-sided specimen will be 1–2% lower than the strength measured with a specific waisted butterfly-shaped specimen solely due to the difference in test specimen shape and volume.

## 1. Introduction

Strength of brittle materials shows size dependency, governed by the distribution of the size of flaws within the volume of the material [1,2,3,4]. The strength of brittle materials is often analysed using the Weibull weakest link theory [5,6,7,8]. The theory assumes that the most critical flaw, i.e., the largest flaw in the tested volume, determines a material’s strength. Weibull proposed a model for the statistical distribution of the probability of failure to analyze the effect of randomly distributed flaws on the strength of the material depending on the tested volume [9]. As the Weibull weakest link theory explained, the strength of brittle materials is increased by decreased materials volume due to a lower probability of finding larger critical flaws in a smaller volume. Thus, when the strength of a brittle material such as carbon fibre composites is measured by mechanical testing, the volume of the test specimen influences the probability of failure, thereby the measured strength. Bullock [10] compared the measured tensile strength of epoxy impregnated carbon fibre tows and unidirectional carbon/epoxy composite test specimens, the latter having 470 times the volume of the tows. Based on the Weibull model, the predicted strength for the composite specimens compared closely to the measured values, supporting the theory. Wisnom et al. [11] performed a series of tensile tests on unidirectional carbon/epoxy composites using test specimens differing in size. The specimens showed a decreasing strength with increasing specimen size, with a reduction of 14% when the specimen volume was increased 512 times. The previous studies primarily focused on analyzing the size effect in the case of specimens having a constant stress state along the specimens.

The tensile strength of a material is a fundamental mechanical property. Its accurate determination requires that test specimens fail in the gauge section, away from the grips [12,13]. However, for high-strength-unidirectional-fibre composites, tensile strength is difficult to determine accurately. Due to these material’s relative low shear strength, test specimens are likely to fail in shear at the grip region. Optimization of specimen geometry, e.g., using non-straight-sided (waisted) specimens, is a common strategy to lower the stresses outside the gauge section and thereby obtain failure of test specimens away from the grips [14,15,16]. Any change in specimen geometry to obtain failure away from the grips will inevitably also lead to a change in the probability of failure due to size dependency. This associated effect of specimen geometry will influence the measured strength. Quantifying the effect of a change of specimen geometry, i.e., its shape and volume, on the probability of failure is essential for determining the actual improvement in measured strength when specimen geometry is optimized.

Based on the Weibull weakest link theory, this paper presents an analytical model that quantifies the stress ratio for the same probability of failure between two test specimens with two different geometries. The ratio of stress for the same probability of failure is a measure of the ratio of measured strength for the two specimens. First, the simple case of two straight-sided specimens with different volumes is analyzed. Second, the more complex case of straight-sided and non-straight-sided specimens is investigated, considering that the stress is not constant along a non-straight-sided specimen. The two comparisons are based on practical relevant specimens widely used for tensile testing of unidirectional fibre composites [14,16,17]. The presented analytical model is, however, general, and the geometrical and materials parameters can be set to analyze the ratio of strength for two specimens with any given shapes and volumes. The model is a practical, valuable tool for determining the actual improvement in measured strength by comparing different specimen geometries taking size scaling into account.

## 2. Motivation

**Figure 1:**
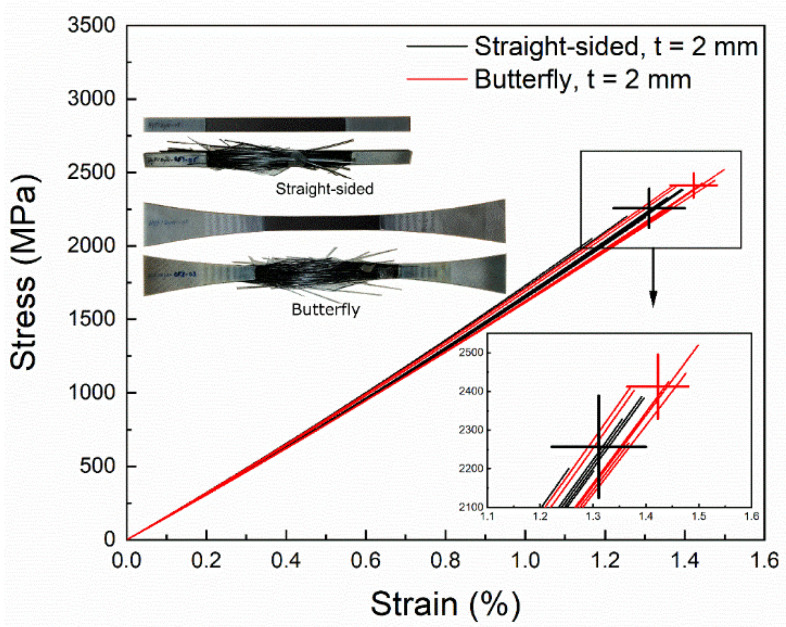
Stress–strain curves of straight-sided (black curves) and butterfly (red curves) test specimens. The crosses (+) at the end of the curves represent standard deviations of failure stress and strain to failure. The inset shows pictures of the test specimens before and after the test.

This study is motivated by a recent experimental study on the effect of specimen geometry on the determined mechanical properties of unidirectional carbon fibre composites under tensile testing by Kumar et al. [16]. Pultruded carbon fibre composites with a thickness of 2 mm were tested. In these composites, all fibres are aligned in one direction, along the test specimens. Comparing butterfly specimens to straight-sided ones, changing specimen geometry led to an increase in failure stress of 7% from (2250 ± 130) MPa to (2400 ± 80) MPa. Figure 1 shows the stress–strain curves for the two test specimen geometries (straight-sided and butterfly), together with pictures of the specimens before and after the test.

The observed improvement in strength of the butterfly specimens was attributed to a lower stress concentration factor than in the straight-sided specimens, which is reducing the risk of failure at the end-tabs of the specimens [16]. However, the difference in test specimen geometry is also leading to a potential size effect, which by itself is giving a difference in strength. Therefore, a study considering size scaling between specimens having different geometries (i.e., shape and volume) is needed to determine the actual improvement in measured strength when specimen geometry is optimized for failure away from the grips. In the present study, it is hypothesized that the observed strength improvement by changing the specimen geometry from straight-sided to butterfly is predominantly due to an improved failure mechanism and not just a size effect.

## 3. The Weibull Model for the Probability of Failure

**Figure 2:**
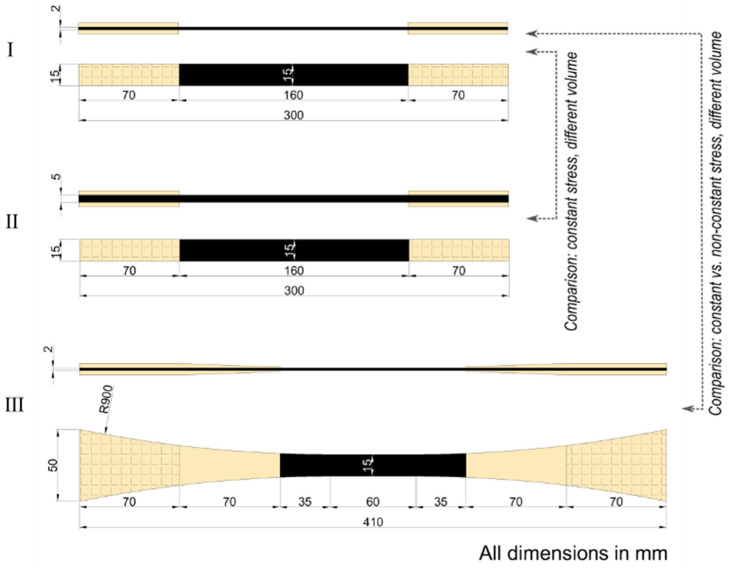
Geometry and dimensions of analyzed test specimens: (I) straight-sided, t=2 mm; (II) straight-sided, t=5 mm; (III) non-straight-sided, butterfly-shaped, t=2 mm. For all three test specimens, the width in the gauge section is 15 mm. Arrows indicate the two comparisons of the stress ratio for the same probability of failure made in the present study. The analyzed part of the specimens is the non-hatched part between the grips.

An analysis of the probability of failure in brittle materials can be performed by the Weibull weakest link model [9]. The probability of failure $P_{F}$ of a material at a stress level σ and volume V can be written as [9]:

(1) $$P_{F}\left(\sigma ,\;V\right)=1-\exp\left(-\frac{V}{V_{0}}{\left(\frac{\sigma }{\sigma_{0}}\right)}^{m}\right)$$

where *m* is the Weibull modulus, $\sigma_{0}$ is the characteristic strength (representing $P_{F}=$ 63%), and $V_{0}$ is the reference material volume having the characteristic strength $\sigma_{0}$. Rearranging the above equation, we obtain:

(2) $$P_{F}\left(\sigma ,\;V\right)=1-\exp\left(-C\;\right)$$

with

(3) $$C=\frac{V\cdot \sigma^{m}}{V_{0}\cdot \sigma_{0}^{m\;}}=V\cdot \sigma^{m}\cdot k\;;\;\;\;k=\frac{1}{V_{0}\cdot \sigma_{0}^{m\;}}$$

where *k* is a material constant. When comparing two test specimens, type I and II, of the same material but with two different volumes $\left(V_{I},\;V_{II}\right)$, (see examples in Figure 2), the probability of failure of the two specimens is the same when $C_{I}=C_{II}$ [9]. In the case of straight-sided test specimens, stress is constant along the specimen length due to the constant cross-sectional area. Thus, for two straight-sided test specimens with different volumes, the ratio of stress in Specimen I ($\sigma_{I}$) to stress in Specimen II ($\sigma_{II}$) that gives the same probability of failure can be derived as:

(4) $$C_{I}=C_{II}$$

(5) $$V_{I}\cdot \sigma_{I}{}^{m}\cdot k=V_{II}\cdot \sigma_{II}{}^{m}\cdot k$$

(6) σIσII=(VIIVI)1m

This expression quantifies the so-called size effect for the strength of brittle materials [18]. The ratio is related to the inverse proportion of tested volumes, and the relationship is governed by the value of the Weibull modulus m.

The ratio of stresses resulting in the same probability for failure in the two different geometries is equal to the ratio of median stresses $\sigma_{I}^{50}$/$\sigma_{II}^{50}$ (or any other fractile). However, experimental tests typically report the mean stress $\bar{\sigma }$ and not the median stress $\sigma^{50}$. For a volume element with constant stress, the following relations are given for the Weibull distribution [19]:

(7) σ50=σ0·(V0V·ln2)1m

(8) σ¯=σ0·(V0V)1m·Γ (1+1m)

where Γ is the gamma function, defined by $\Gamma \left(x\right)=\int_{0}^{\infty }\exp\left(-u\right)\cdot u^{x-1}du$ [20].

Based on the two expressions in Equations (7) and (8), it can be seen that for a given set of material parameters ($m,\;V_{0},\sigma_{0}$), the ratio between stress in Specimen I and stress in Specimen II in terms of the median and the mean coincides:

(9) $$\frac{\sigma_{I\;50}}{\sigma_{II\;50}}=\frac{{\bar{\sigma }}_{I}}{{\bar{\sigma }}_{II\;}}$$

This shows that the ratio of stress for the same probability of failure is also a measure of the ratio of measured mean strength for two specimens.

The relationship between the Weibull modulus m and the coefficient of variation (cv=stdv./mean) can be approximated by a simple relation [19]:

(10) m≃1.2/cv

Equation (10) makes it possible to approximate the Weibull modulus m for a given material from a series of strength measurements where the mean and the standard deviations are determined.

### 3.1. Test Specimens

Figure 2 shows the geometry and dimensions of the three test specimens analyzed in the present study. These test specimens are widely used to measure the strength of unidirectional fibre composite materials [14,16,17]. Here are the overall specifications of the analyzed three test specimens:

- Straight-sided specimen, based on standard ISO 527-5:2009 [21]; thickness t=2 mm, cross-sectional area $A=30{\;\mathrm{mm}}^{2}$, tested volume $V=4800{\;\mathrm{mm}}^{3}$.
- Straight-sided specimen, based on standard ISO 527-5:2009 [21]; thickness t=5 mm, cross-sectional area $A=75{\;\mathrm{mm}}^{2}$, tested volume $V=12,000{\;\mathrm{mm}}^{3}$.
- Non-straight-sided specimen, butterfly-shaped, designed at DTU Wind Energy [14]; thickness t=2 mm, cross-sectional area A in the range $30–55\;{\mathrm{mm}}^{2}$, tested constant stress volume $V=1800{\;\mathrm{mm}}^{3}$, tested non-constant stress volume $V=9800{\;\mathrm{mm}}^{3}$.

Figure 2 shows the two comparisons of the ratio of stress for the same probability of failure that are made in the present study. First, the stress ratio is determined for the two constant stress specimens, I and II, having different volumes. Second, the stress ratio is determined for the constant stress Specimen I and the non-constant stress Specimen III, having different shapes and volumes. An open-access Jupyter notebook for analyzing and plotting the two comparative cases is available in reference [22].

### 3.2. Comparison between Constant Stress Specimens

The stress ratio for the same probability of failure for two constant stress specimens with different test volumes can be directly analyzed using Equation (6). For a comparison of the straight-sided Specimens I and II, where only the thickness differs between the specimens, the stress ratio is given as:

(11) σIσII=(tII·wII·lIItI·wI·lI)1m=(tIItI)1m

where t,w, and l are the tested volume’s thickness, width, and length.

**Figure 3:**
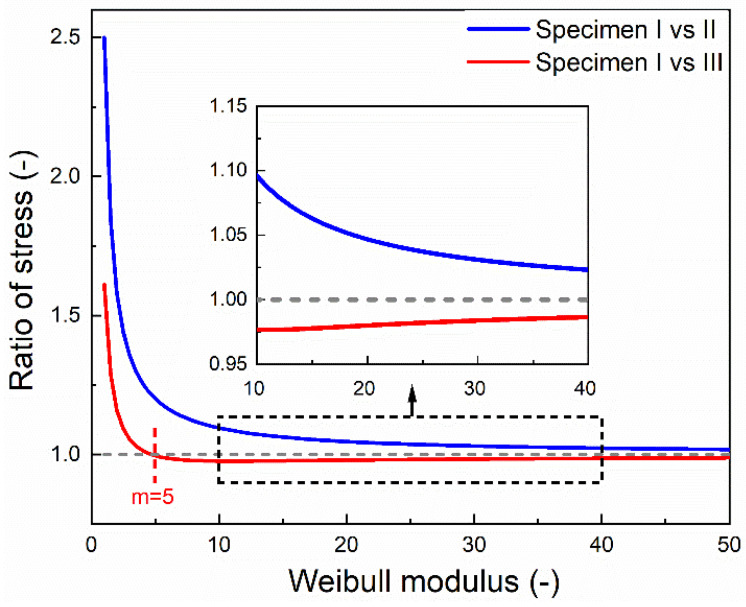
The ratio of stress for the same probability of failure as a function of the Weibull modulus. Shown are the two analyzed comparisons between test Specimens I and II (blue curve) and test Specimens I and III (red curve).

The blue curve in Figure 3 shows the stress ratio arising from a difference in tested volumes of Specimen I and II as a function of the Weibull modulus m (from 1 to 50). For the lowest m=1, the ratio of stress becomes equal to the ratio of thicknesses of the specimens ($\frac{\sigma_{I}}{\sigma_{II}}=\frac{5}{2}=2.5$). With an increasing m value, the ratio of stress tends to approach unity. Thus, with an increasing m value, the size effect in the specimen diminishes. For unidirectional carbon fibre composites, values of m have been reported in the range of 10−40 [11,18]. In a study by the authors where unidirectional carbon fibre composites were tested using protected X-butterfly specimens [16], the coefficient of variation was found to be cv=0.042, and m can then be calculated from Equation (10) to be ≈28. For m∈[10; 40] (typical for unidirectional carbon composites), it can be seen in the zoomed insert of Figure 2 that $\frac{\sigma_{I}}{\sigma_{II}}\in \left[1.02;1.09\right]$. Thus, the strength measured with Specimen I will be in the range of 2−9% above the strength measured with Specimen II due to the difference in tested volume.

### 3.3. Comparison between Constant vs. Non-Constant Stress Specimens

In the non-straight-sided Specimen III (see Figure 2), the stress varies along the length of the specimen, with the highest stress occurring in the $60\times 15\times 2\;{\mathrm{mm}}^{2}$ central constant stress region (i.e., the gauge section).

**Figure 4:**
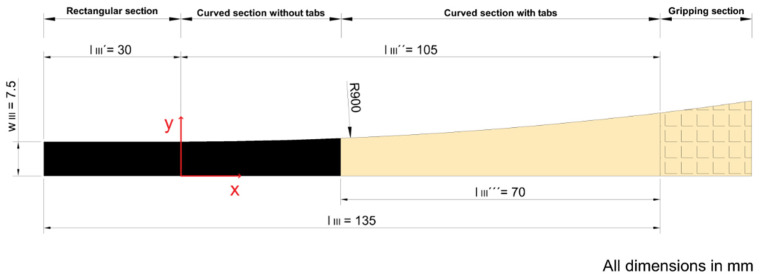
The geometrical and notational basis for analysis of Specimen III.

The geometrical and notational basis for the analysis of Specimen III is shown in Figure 4. Due to symmetry, only one-quarter of the specimen is taken into consideration. The tested volume is divided into three sections: (a) central rectangular section, (b) curved section without tabs, and (c) curved section with tapered tabs. The origin of the x–y coordinate system is at the start of the first curved section. The width w(x) of the specimen along the length $l_{III}$ of the specimen can be expressed as:

(12) $$w\left(x\right)=\{\begin{array}{c} w_{III}\;\\ w_{III}+R-\sqrt{R^{2}-x^{2}} \end{array}$$

for $x\in \left[-\ell_{III}^{\prime };0\right]$ (central rectangular section);

for $x\in \left[0;\ell_{III}^{\prime \prime }\right]$ (curved sections, without and with tabs);

where $w_{III}$ is the width at the center of the specimen, and R is the radius of curvature of the curved sections of the specimen.

Considering then force equilibrium between the central rectangular section and the two curved sections in the specimen:

(13) $$\sigma_{III}\cdot t\cdot w_{III}=\left[E_{C}\cdot \varepsilon \left(x\right)\cdot t+E_{tab}\cdot \varepsilon \left(x\right)\cdot h\left(x\right)\right]\;w\left(x\right)\;\mathrm{for}\;x\in \left[0;\ell_{III}^{\prime \prime }\right]$$

where $E_{C}$,$\;E_{tab}$, t, h(x), and ε(x) is the stiffness of the test laminate, the stiffness of the tab material, the thickness of the test laminate, the thickness of the tab as a function of x, strain in the specimen as a function of x, respectively. The parameter $\sigma_{III}$ is the stress value in the central rectangular region (i.e., the gauge section).

Using Equation (13), the strain distribution ε(x) in the two curved sections of the specimen can be written as:

(14) $$\varepsilon \left(x\right)=\frac{\sigma_{III}\cdot t\cdot w_{III}}{\left[E_{C}\cdot t+E_{tab}\cdot h\left(x\right)\right]\;w\left(x\right)}$$

The stress distribution in the non-constant stress part of the specimen as a function of the stress value in the gauge section ($\sigma_{III}$) can then be established using Hooke´s law:

(15) $$\sigma \left(x\right)=E_{C}\cdot \varepsilon \left(x\right)=\sigma_{III}\cdot \frac{w_{III}}{w\left(x\right)}\;\;\frac{E_{C}\cdot t}{E_{C}\cdot t+E_{tab}\cdot h\left(x\right)}$$

where σ(x) and ε(x) is assumed only to be a function of the longitudinal position, x.

One limitation in the present study is that the stress state is assumed to be constant across any given cross section (width direction). However, Korkiakoski et al. [14] found some variation of the stresses in the width direction in specimen geometry III. Nevertheless, σ(x) in Equation (15) will be equal to the mean value of this stress variation. The influence of stress variation in the width direction is expected to have only a marginal influence [14]. This marginal influence can be investigated by using the actual stress variation obtained from a finite element model.

Using Equation (15), the stress distribution for the two curved sections can be separated, and it can be written as:

(16) $$\sigma \left(x\right)=\sigma_{III}\cdot \left(\frac{w_{III}}{w\left(x\right)}\cdot f\left(x\right)\right);\;f\left(x\right)=\{\begin{array}{c} 1\\ \frac{1}{1+\left(\left(\frac{E_{tab}}{E_{C}}\right).\left(\frac{h\left(x\right)}{t}\right)\right)} \end{array}\begin{array}{c} \;\mathrm{for}\;x\in \left[0;l_{III}^{\prime \prime }-l_{III}^{\prime \prime \prime }\right]\;\\ \;\mathrm{for}\;x\in \left[l_{III}^{\prime \prime }-l_{III}^{\prime \prime \prime };l_{III}^{\prime \prime }\right] \end{array}$$

The varying linear thickness of the tapered tab can be represented as:

(17) $$h\left(x\right)=\left(\frac{h_{0}}{l_{III}^{\prime \prime \prime }}\right)\cdot x+h_{0}\cdot \left(1-\frac{l_{III}^{\prime \prime }}{l_{III}^{\prime \prime \prime }}\right)$$

where $h_{0}$ is the maximum thickness of the tab at the end of the gripping region. This can be rewritten as:

(18) $$\frac{h\left(x\right)}{t}=\frac{h_{0}}{t}\cdot \left(\frac{x+l_{III}^{\prime \prime \prime }-l_{III}^{\prime \prime }}{l_{III}^{\prime \prime \prime }}\right)$$

where t is the thickness of the test laminate. Having established the above equations for Specimen III, an infinitesimal segment of the specimen with test laminate thickness t, width w(x), and length dx can be considered. Based on this small segment, $C_{III}$, from Equation (3), can be written as:

(19) $$dC_{III}=t\cdot w\left(x\right)\cdot dx\cdot \sigma {\left(x\right)}^{m}\cdot k$$

By substituting the expression for σ(x) from Equation (16), the following expression can be obtained:

(20) $$dC_{III}=t\cdot w\left(x\right)\cdot dx\cdot {\left(\sigma_{III}\cdot \left(\frac{w_{III}}{w\left(x\right)}\cdot \;f\left(x\right)\right)\right)}^{m}\cdot k$$

(21) $$dC_{III}=t\cdot w_{III}^{m}\cdot \sigma_{III}^{m}\cdot w{\left(x\right)}^{1-m}\cdot f{\left(x\right)}^{m}\cdot dx\cdot k$$

Integrating Equation (21) over the length of the specimen, an expression for $C_{III}$ can be found:

(22) $$C_{III}=t\cdot w_{III}^{m}\cdot \sigma_{III}^{m}\cdot \left[\int_{-l_{III}^{\prime }\;}^{l_{III}^{\prime \prime }\;}{(w\left(x\right)}^{1-m}\cdot f{\left(x\right)}^{m}\cdot dx\right]\cdot k$$

Now, aiming for the same probability of failure, $C_{I}$ = $C_{III}$, we obtain:

(23) $$t\cdot w_{I}\cdot l_{I}\cdot \sigma_{I}{}^{m}\cdot k=t\cdot w_{III}^{m}\cdot \sigma_{III}^{m}\cdot \left[\int_{-l_{III}^{´}\;}^{l_{III}^{\prime \prime }\;}{(w\left(x\right)}^{1-m}\cdot f{\left(x\right)}^{m}\cdot dx\right]\cdot k$$

(24) $${\left(\frac{\sigma_{I}}{\sigma_{III}}\right)}^{m}=\frac{w_{III}}{w_{I}}\cdot \frac{1}{l_{I}}\int_{-l_{III}^{\prime }}^{l_{III}^{\prime \prime }\;}{\left(\frac{w\left(x\right)}{w_{III}}\right)}^{1-m}\cdot f{\left(x\right)}^{m}\cdot dx$$

By substituting the expression for w(x) and f(x), the following expression can be obtained for the ratio of stress in the gauge section of straight-sided and non-straight-sided specimens for the same probability of failure.

(25) σIσIII=[wIIIwI]1m·[1lI∫0 lIII″−lIII‴(wIII+R−R2− x2wIII)1−mdx    + 1lI∫lIII″−lIII‴ lIII″ (wIII+R−R2− x2wIII)1−m(1+Etab·h0·(x+lIII‴−lIII″)EC·t·lIII‴ )−mdx + lIII'lI]1m

For Specimen I, the dimensions $l_{I}=80\;\mathrm{mm}$ and $w_{I}=7.5\;\mathrm{mm}$. For Specimen III, the dimensions are shown in Figure 4. Thickness t=2 mm is the same for the two specimens. For Specimen III, the maximum tab thickness $h_{0}=2\;\mathrm{mm}$. Calculations are performed for a unidirectional carbon composite material with stiffness $E_{C}=153\;\mathrm{GPa}\;$[16] and with tabs of a biaxial glass composite material with an axial stiffness $E_{tab}\;$= 15 GPa [23]. Figure 3 shows the determined ratio of stress arising from the difference in shape and volume of Specimens I and III as a function of the Weibull modulus m (from 1 to 50). For m=1, i.e., very large scatter in strength, the ratio of stress is equal to 1.61, and this can be seen from Equation (25) to be approximately equal to the ratio of the length of the specimens ($\frac{l_{III}}{l_{I}}=1.69$), neglecting the small contribution of the low stiffness tabs. In the case of m=1, the equation becomes independent of the varying width w(x) along the length of Specimen III. At m=5, there is a transition in the ratio of stress from above unity to below unity. Thus, for the specific case, for m<5, the strength measured with the straight-sided Specimen I will be higher than that measured with the non-straight-sided Specimen III, and vice versa for m>5. This shift is caused by the constant stress volume of Specimen III being smaller than the test volume of Specimen I, while the total test volume of Specimen III is larger than the test volume of Specimen I. If the entire test volume of Specimen III´s were smaller than the test volume of the constant stress Specimen I, then the ratio would be $\frac{\sigma_{I}}{\sigma_{III}}<1$ for all m values. With increasing m values above 5, the contribution of varying width w(x) and the difference in specimen length $l_{I}$ and $l_{III}$ becomes smaller. The ratio of stress approaches 1, meaning that the strength measured by the two specimens becomes the same. For m∈[10; 40] (typical for unidirectional carbon composites), it can be seen in the zoomed insert of Figure 3 that $\frac{\sigma_{I}}{\sigma_{III}}\in \left[0.98;0.99\right]$. Thus, the strength measured with Specimen I will be 1–2% lower than that measured with Specimen III due to the difference in tested shape and volume. The strength measured using Specimen I was 7% lower than Specimen III. The improvement in measured strength observed for Specimen III was attributed to its low curvature and long tapered end-tabs, reducing the risk of failure at end-tabs [16]. The present study shows that the experimentally determined 7% improvement in strength is more significant than the 1–2% contribution solely due to the scale effect coming from the difference in tested shape and volume of Specimens I and III.

## 4. Conclusions

Based on the Weibull weakest link theory, this paper presents an analytical model for analyzing the ratio of stress for the same probability of failure between two test specimens. The model is a valuable tool to quantify the effect of a change of specimen geometry (i.e., its shape and volume) on the probability of failure. This is essential for determining the actual improvement in measured strength when specimen geometry is optimized. When comparing constant stress specimens, the ratio of stress remains above unity for all ranges of m values, which describes the well-known size effect for the strength of materials. However, for this specific case, when comparing constant vs. non-constant stress specimens, due to the combined contribution of shape and volume of a non-straight-sided specimen, at a given m value, there is a transition in the ratio of stress from above unity to below unity. For one specific case of constant vs. non-constant stress specimens of unidirectional carbon fibre composites with Weibull m values in the range 10–40, the strength measured with the straight-sided specimen is determined to be 1–2% lower than the strength measured with a butterfly-shaped specimen solely due to the difference in tested shape and volume.

## Data Availability

An open-access Jupyter notebook for analyzing and plotting the two comparative cases is available in reference [22].
